# A ROUTINE BLOOD PARAMETER BIOLOGICAL AGE COMPOSITE IS ASSOCIATED WITH FRAILTY IN THE BERLIN AGING STUDY II (BASE-II)

**DOI:** 10.1093/geroni/igac059.2452

**Published:** 2022-12-20

**Authors:** Ilja Demuth, Valentin Vetter, Johanna Drewelies, Sandra Duezel, Dominik Spira, Elisabeth Steinhagen-Thiessen, Lars Bertram, Denis Gerstorf

**Affiliations:** Charité Universitätsmedizin Berlin, Berlin, Berlin, Germany; Charité - Universitätsmedizin Berlin, Berlin, Berlin, Germany; Humboldt University Berlin, Berlin, Berlin, Germany; Max-Planck-Insitute for human developement, Berlin, Berlin, Germany; Charité - Universitätsmedizin Berlin, Berlin, Berlin, Germany; Charité - Universitätsmedizin Berlin, Berlin, Berlin, Germany; University of Lübeck, Lübeck, Schleswig-Holstein, Germany; Humboldt Universität zu Berlin, Berlin, Berlin, Germany

## Abstract

High-quality biomarkers are needed to predict age-related phenotypes and evaluate health-related interventions. Telomere length has been known as a biomarker of aging for some time, however, its potential for clinical use is limited. Epigenetic age estimators and biological age (bioage) composites have been developed and refined over the past decade and their evaluation is ongoing. Aim of the current study was to further evaluate a recently developed bioage composite based on 12 routine laboratory blood parameters which have been shown to individually predict mortality risk. To this end, we assessed the relationship between the bioage composite, frailty and a number of functional assessments. The bioage composite was calculated for participants of the Berlin Aging Study II (BASE-II) aged ≥ 60 years (N=1,278, mean age 68.6 ±3.7 years, 53.3% women). Frailty was operationalized by Fried’s five-point frailty score. Functional capacity was assessed with a battery of tests including the Tinetti mobility test, finger-floor distance (FFD), Mini-Mental State Examination (MMSE), Center for Epidemiologic Studies Depression Scale (CES-D), activities of daily living (ADL) and instrumented ADL (IADL). In covariate adjusted regression analyses the deviation of the bioage composite from chronological age was significantly associated with frailty (beta = -0.014, p= 0.014), the CES-D and ADL in men, and with the FFD in women. These results will be discussed with respect to their clinical relevance and compared to previously reported BASE-II data on the relationship between frailty, functional assessments and the biomarkers of aging derived from epigenetic clock algorithms and telomere length.

